# Assessing the pathogenicity of *BRCA1/2* variants of unknown significance: Relevance and challenges for breast cancer precision medicine

**DOI:** 10.3389/fonc.2022.1053035

**Published:** 2023-01-18

**Authors:** Elisa De Paolis, Ida Paris, Bruno Tilocca, Paola Roncada, Laura Foca, Giordana Tiberi, Tatiana D’Angelo, Francesco Pavese, Margherita Muratore, Luisa Carbognin, Giorgia Garganese, Riccardo Masetti, Alba Di Leone, Alessandra Fabi, Giovanni Scambia, Andrea Urbani, Daniele Generali, Angelo Minucci, Concetta Santonocito

**Affiliations:** ^1^ Clinical Chemistry, Biochemistry and Molecular Biology Operations (UOC), Fondazione Policlinico Universitario A. Gemelli IRCCS, Rome, Italy; ^2^ Department of Basic Biotechnological Sciences, Intensivological and Perioperative Clinics, Catholic University of Sacred Heart, Rome, Italy; ^3^ Division of Oncological Gynecology, Department of Women’s and Children’s Health, Fondazione Policlinico Universitario A. Gemelli IRCCS, Rome, Italy; ^4^ Department of Health Science, University “Magna Graecia” of Catanzaro, Catanzaro, Italy; ^5^ Gynaecology and Breast Care Center, Mater Olbia Hospital, Olbia, Italy; ^6^ Dipartimento Universitario Scienze della Vita e Sanità Pubblica, Sezione di Ginecologia ed Ostetricia, Università Cattolica del Sacro Cuore, Rome, Italy; ^7^ Unit of Precision Medicine in Breast Cancer, Scientific Directorate, Department of Woman and Child Health and Public Health, Fondazione Policlinico Universitario A. Gemelli, IRCCS, Rome, Italy; ^8^ Department of Medical, Surgery and Health Sciences, University of Trieste, Trieste, Italy; ^9^ Departmental Unit of Molecular and Genomic Diagnostics, Fondazione Policlinico Universitario A. Gemelli IRCCS, Rome, Italy

**Keywords:** breast cancer, triple negative early-stage breast cancer, BRCA1/2, variants of unknown significance, precision medicine

## Abstract

**Introduction:**

Breast cancer (BC) is the leading cause of cancer-related death in women worldwide. Pathogenic variants in BRCA1 and BRCA2 genes account for approximately 50% of all hereditary BC, with 60-80% of patients characterized by Triple Negative Breast Cancer (TNBC) at an early stage phenotype. The identification of a pathogenic BRCA1/2 variant has important and expanding roles in risk-reducing surgeries, treatment planning, and familial surveillance. Otherwise, finding unclassified Variants of Unknown Significance (VUS) limits the clinical utility of the molecular test, leading to an “imprecise medicine”.

**Methods:**

We reported the explanatory example of the BRCA1 c.5057A>C, p.(His1686Pro) VUS identified in a patient with TNBC. We integrated data from family history and clinic-pathological evaluations, genetic analyses, and bioinformatics in silico investigations to evaluate the VUS classification.

**Results:**

Our evaluation posed evidences for the pathogenicity significance of the investigated VUS: 1) association of the BRCA1 variant to cancer-affected members of the family; 2) absence of another high-risk mutation; 3) multiple indirect evidences derived from gene and protein structural analysis.

**Discussion:**

In line with the ongoing efforts to uncertain variants classification, we speculated about the relevance of an in-depth assessment of pathogenicity of BRCA1/2 VUS for a personalized management of patients with BC. We underlined that the efficient integration of clinical data with the widest number of supporting molecular evidences should be adopted for the proper management of patients, with the final aim of effectively guide the best prognostic and therapeutic paths.

## 1 Introduction

Breast cancer (BC) is the most frequently diagnosed cancer in women worldwide. Mutations in *BRCA1* and *BRCA2* (*BRCA*) genes accounted for 50% of hereditary BC and up to 10% of the total BC cases (1, 2). Triple-negative BC (TNBC) at an early stage accounts for 15% of all BC, and it is associated with poor long-term outcomes, compared with other BC subtypes. TNBC is enriched for germline *BRCA* mutations, providing a rational basis for the use of the molecular test as a biomarker to identify patients suitable for molecular target treatment and preventive planning (3). The *BRCA* tumor suppressor genes preserve the DNA integrity, allowing the double-strand DNA breaks repairing by the homologous recombination (HR) system (4). Consequently, the impairment of the BRCA proteins activity due to loss-of-function (LoF) pathogenic variants can markedly compromise the repair system effectiveness, leading to an increased individual’s likelihood of developing cancer (5). In addition, germline deleterious *BRCA* alterations are associated with increased risk for several types of cancer, including ovarian, pancreatic, prostate, and melanoma (6). To date, genetic testing for *BRCA* genes represents a well-known strategy aimed at guiding the clinical management of BC and the preventive paths of patients’ relatives (7). Guidelines for the management of *BRCA* pathogenic variant carriers recommend consideration of risk-reducing procedures or prophylactic surgeries (8). However, only the finding of a *BRCA* genetic variant with a clear deleterious significance represents a clinical actionable information. On the contrary, the identification of unclassified *BRCA* variants of unknown significance (VUSs) limits the clinical utility of the molecular test being related to issues for proper risk calculation (9).

VUSs are mainly missense variants that cannot be definitely classified as pathogenic or not, due to the insufficiency of experimental and clinical data. About this, the American College of Medical Genetics and Genomics (ACMG) outlined: “Efforts to resolve the classification of the variant as pathogenic or benign should be undertaken. While this effort to reclassify the variant is underway, additional monitoring of the patient for the disorder in question may be prudent” (10). To evaluate the VUS significance and to speculate about its classification, several parameters and types of evidence can be taken into account: 1) co-segregation analysis of the VUS with the disease in multiple and independent families; 2) frequency of the VUS in affected cases and controls; 3) personal and family history of VUS carriers; 4) co-occurrence of another high-risk mutation in the affected patients; 5) indirect information about amino acid interspecies conservation and prediction of the substitution impact on the protein structure and/or function; and 6) *in vitro* functional assays (11, 12). It is acknowledged that the maximized integration of these lines of evidence allows the understanding of the disease-causality and solves the “imprecise medicine” related to VUS identification (13). Although this approach is often pivotal, it is unlikely to apply in routine clinical context and it may not fit with the short-term goal of targeted treatment. In fact, identifying the VUSs that really affect protein structure and/or function, causing the observed patient’s phenotype, is time and labor consuming (14). In our large cohort of more than 1,000 *BRCA* germline tests performed annually, a frequency of approximately 5% resulted in a VUS identification that raises troublesome questions for both genetics and oncologist professionals. According to the ACMG guidelines, VUS finding should be interpreted as non-informative result and should not directly influence cancer management. At the same time, individualized screening and prevention strategies are advantageous in such cases (15).

The aim of this study is to underline the need of a dedicated clinical path for patients’ carriers of VUS. To achieve this, we described our ongoing efforts to assess pathogenicity of uncertain variants, reporting an explanatory example belonging from our Institution. The tailored molecular characterization strategy here reported represents an integration of clinical data with the widest number of supporting molecular evidence. This approach does not compromise the turnaround time of the test and supports a multi-disciplinary investigation, peculiarity of the current precision medicine.

## 2 Materials and methods

### 2.1 Family history and clinic-pathological evaluation

Approval from the Ethical Committee our Institution Fondazione Policlinico Universitario “A. Gemelli” was obtained (protocol ID 5111). We described the case of a Ukranian 65-year-old patient diagnosed with TNBC at the Medical Breast Unit of the Fondazione Policlinico Universitario “A. Gemelli” in Rome (Italy). On October 2020, her x-ray mammography revealed a breast mass in upper outer right quadrant of 26 mm. Breast ultrasound and magnetic resonance confirmed a single lesion of 25 mm without axillary metastases. After a fine-needle aspiration biopsy, the patient underwent right quadrantectomy with sentinel lymph node biopsy on December 2020 with diagnosis of invasive ductal TNBC, pT2 (30 mm) with pN0 (0/2 sn), estrogen receptor of 0%, progesterone receptor of 0%, androgen receptor of 0%, ki67 of 90%, and human epidermal growth factor receptor 2 (HER2) of 0. Radiological staging was negative for distant metastases. From February to June 2021, the patient received four courses of adjuvant chemotherapy with EC90 q21, followed by 12 weekly paclitaxel doses. She was subsequently treated with adjuvant radiotherapy. On the family history evaluation, the patient presented a suspicious familiarity for BC and *BRCA*-related cancers: one brother died for pancreatic cancer at young age, one daughter alive with a previous adenocarcinoma of the uterine cervix (35 years old), and three nieces affected by BC of which two lives. It was not possible to collect information about the other family members. Oncologist addressed the proband to *BRCA* genetic testing performed at the Departmental Unit of Molecular and Genomic Diagnostics of the Fondazione Policlinico Universitario “A. Gemelli” in Rome (Italy) in February 2021. The genetic screening was offered also to the cancer affected proband’s daughter, whereas an affected niece performed the *BRCA* testing in a tertiary referral center. Both proband’s parents are deceased so we were not able to check the *BRCA* status and relevant clinical information were not available. Patients signed written informed consent before the genetic test and received oncologist and genetic counseling before and after the genetic test.

### 2.2 *BRCA* genetic test and high-risk multi-gene evaluation

Blood samples were obtained from each patient using Greiner BLO-ONE Vacuum EDTA Tubes. Germline DNA extractions were performed using the QIAmp DNA Mini kit (Qiagen) on the QIAcube (Qiagen) automated platform, according to the manufacturer’s instructions. The Next Generation Sequencing (NGS) Devyser *BRCA* assay (Devyser) was at first offered for the target molecular evaluation of *BRCA1* (NM_007294) and *BRCA2* (NM_000059) genes in the proband and in her daughter. The amplicon-based Devyser *BRCA* kit covers all the coding regions and the exons boundaries of *BRCA* genes, starting from 10 ng of DNA. According to the manufacturer’s protocol, the final library pool was quantified using the Qubit dsDNA HS fluorimetric assays (Life Technologies) and sequenced in paired-ends reads mode (2X251 cycles) with FastQ only analysis workflow on the Illumina MiSeq^®^ platform (Illumina).

Given the finding of a VUS, a multi-gene evaluation was afterward performed. In particular, we adopted the 26-gene NGS panel named Sophia™ Hereditary Cancer Solution (HCS) (Sophia Genetics) that covers the coding regions and splicing junctions of the following genes: *ABRAXAS1* (NM_139076.3), *ATM* (NM_000051.3), *APC* (NM_001127511.3), *BARD1* (NM_000465.3), *BRCA1* (NM_007294.3), *BRCA2* (NM_000059.3), *BRIP1* (NM_032043.2), *CDH1* (NM_004360.5), *CHEK2* (NM_001005735.1), *EPCAM* (NM_002354.3), *MLH1* (NM_000249.4), *MRE11* (NM_005591.4), *MSH2* (NM_000251.3), *MSH6* (NM_000179.3), *MUTYH* (NM_001350650), *NBN* (NM_002485.5), *PALB2* (NM_024675.3), *PIK3CA* (NM_006218.4), *PMS2* (NM_000535.7), *PTEN* (NM_001304717.5), *RAD50* (NM_005732.4), *RAD51C* (NM_058216.2), *RAD51D* (NM_002878.3), *STK11* (NM_000455.4), *TP53* (NM_000546.5), and *XRCC2* (NM_005431.1). In the HCS sequencing workflow, NGS libraries were prepared starting from 100 ng of DNA using a KAPA HyperPlus library preparation kit (Roche Diagnostics), according to the manufacturer’s protocol. DNA fragments were generated using an enzymatic fragmentation step. The three subsequent enzymatic steps—end-repair, A-tailing, and ligation to Illumina adapters—were performed. A capture-based target enrichment was carried out on pooled libraries. Quantitation of the final pool of libraries was performed using the Qubit dsDNA HS fluorimetric assays (Life Technologies). Quality control of fragments size was assessed using DNA ScreenTape analysis (4200 TapeStation system, Agilent Technologies). Sequencing run was performed in paired-ends reads mode with FastQ only analysis workflow on the Illumina MiSeq^®^ NGS platform (Illumina).

Both molecular approaches investigated single-nucleotide variants, insertions and deletions (*indels*), and copy number variation (CNV) events in the analyzed genes.

### 2.3 *BRCA* VUS interpretation and *in silico* investigation

Sequencing FastQ data belonging from *BRCA* genes analysis were analyzed by CE-IVD Amplicon Suite Software (SmartSeq), whereas sequencing results belonging from multi-gene panel were analyzed using SophiaDDM platform (Sophia Genetics). The bioinformatic CNV prediction was performed by analysing the coverage levels of the target regions across samples, with the resolution of single exon. The variant interpretation and classification were obtained according to the integration of several online databases (last access September 2022) including dbSNP (https://www.ncbi.nlm.nih.gov/snp/), 1000 Genomes (http://www.internationalgenome.org/), GnomAD (https://gnomad.broadinstitute.org/), ClinVar (http://www.ncbi.nlm.nih.gov/clinvar/), Leiden Open Variation Database (LOVD) (https://www.lovd.nl/), *BRCA* Exchange (https://brcaexchange.org/variant/582350), Human Gene Mutation Database (http://www.hgmd.cf.ac.uk/ac/index.php), Breast Cancer Information Core (BIC) (https://research.nhgri.nih.gov/projects/bic/), and Evidence-based Network for the Interpretation of Germline Mutation alleles (ENIGMA) Consortium (http://enigmaconsortium.org/).

For the *in silico* bioinformatic prediction of pathogenicity, the VarSome (https://varsome.com/) tool was systematically used for VUS detected in our patients. This tool integrates available clinical information related to the variant with literature and epidemiological data, combining evidence from 18 multiple computational algorithms as Poly-Phen-2 (http://genetics.bwh.harvard.edu/pph2/), Provean (http://provean.jcvi.org/seq_submit.php), Sift (https://sift.bii.a-star.edu.sg/www/SIFT_seq_submit2.html), and MutationTaster (http://www.mutationtaster.org/. Moreover, we checked the prediction of pathogenicity using the PRIOR software (http://priors.hci.utah.edu/PRIORS/BRCA/viewer.php?gene=BRCA1) and the InterVar tool for the clinical interpretation according to the ACMG/AMP 2015 guideline (https://wintervar.wglab.org/results.php). In addition, the Clustal Omega (https://www.ebi.ac.uk/Tools/msa/clustalo/) and the Align GVGD (http://agvgd.hci.utah.edu/agvgd_input.php) multiple sequence alignment programs were queried to evaluate the amino acid conservation among species at the variant site and to estimate the impact of the missense change. Furthermore, the molecular stability of the wild-type and the mutant proteins was computed through DynaMut (http://biosig.unimelb.edu.au/dynamut/). This tool relies on the three-dimensional (3D) structures of the proteins available on the PBD database (https://www.rcsb.org/) and performs the molecular stability analysis by considering the physical and chemical interactions occurring among the amino acid residues of the protein, including the amino acidic sequence, the distance between residues, and the protein folding. Each of the predicted interactions is accounted as a molecular twist force that either stabilizes or destabilizes the whole molecule. The sum of all computed interaction reveals the overall thermodynamic stability of the queried sequence (16). Finally, protein–protein interaction (PPI) trend for the wild-type and mutant protein was calculated by using both PSOPIA (https://mizuguchilab.org/PSOPIA/index.html) and Tri-Tool (http://www.vin.bg.ac.rs/180/tools/tfpred.php) against a list of target proteins selected as the most likely candidate of interaction with *BRCA1* (17, 18). PSOPIA is a bioinformatic tool that predicts interaction between pairs of protein given their amino acidic sequences and without prior structural information. This tool computes PPIs by evaluating 1) sequence similarities to a known interacting protein pair, 2) statistical propensities of domain pairs observed in interacting proteins, and 3) define PPI network to sum edge weights along the shortest path between homologous proteins (19).

Tri-Tool predicts PPI in a sequence-based manner. The tool is trained on a dataset of over 24,400 transcriptional regulation interactions validated on human-tailored experiments, resulting in a powerful tool for biomedical investigations (20).

## 3 Results

**Figure 1:**
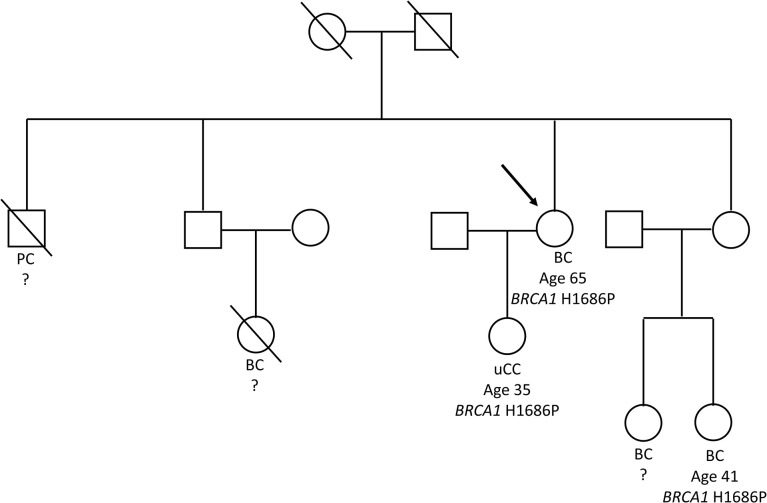
Tree of the Ukranian family bearing the *BRCA1* p.His1686Pro variant. The figure shows the family tree with details about *BRCA1* genotype and cancers occurrence. The proband was depicted with an arrow. BC, breast cancer; PC, pancreatic cancer; uCC, adenocarcinoma of the uterine cervix.

**Figure 2:**
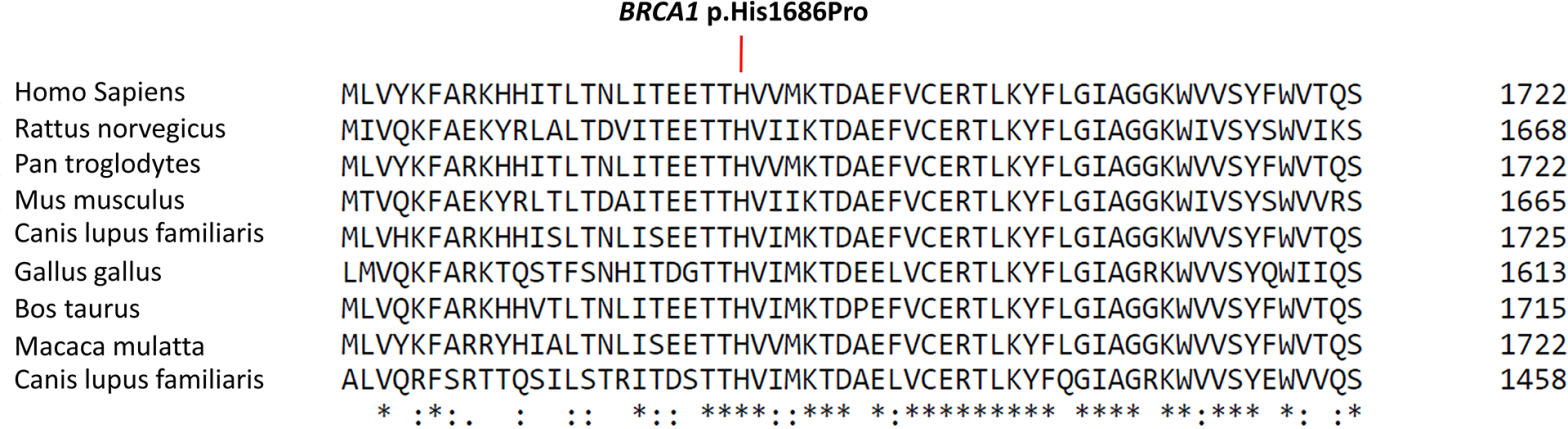
Protein sequence alignment of *BRCA1* region of interest. The figure shows the Cluscal Omega multi-sequence alignment of part of the BRCA1 N-terminal BRCT domain showing the conservation of the THV motif among the species. Location of the BRCA1 p.His1686Pro variant is detailed in the box. An asterisk “*” indicates that the amino acid residue is fully conserved among the selected species, a colon “:” means that a conservative substitution between residues with similar properties has been observed in the alignment, and a period “.” describes a semi-conservative substitution between residues with weakly similar proprieties (https://www.ebi.ac.uk/Tools/msa/clustalo/).

**Figure 3:**
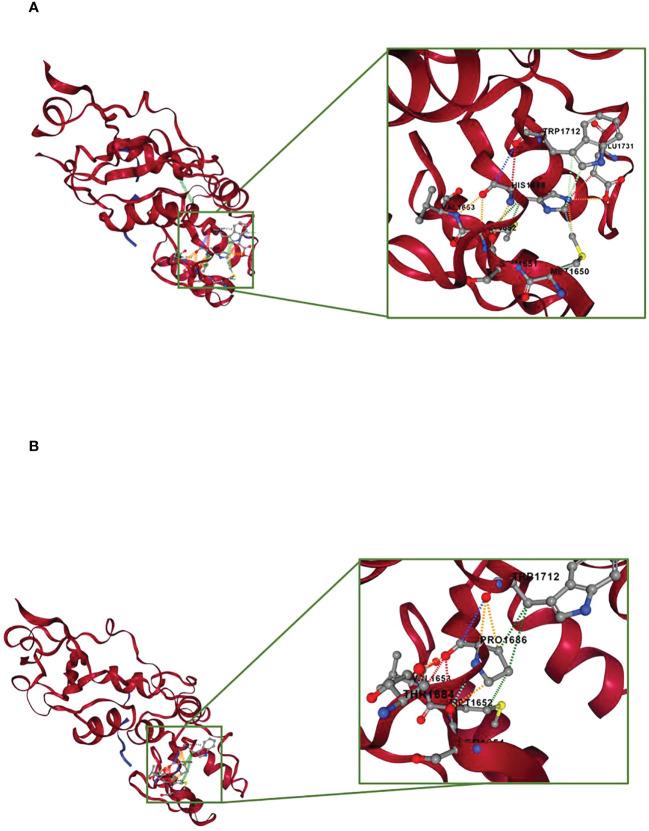
Prediction of molecular stability by DynaMut. The protein portion concerned by the point mutation is zoomed and depicted in the framed box. **(A)** Structural prediction of the BRCA1 wild-type protein. **(B)** Structural prediction of the BRCA1 p.His1686Pro protein. The interactions occurring between the p.His1686 residue and the neighbor residues are showed as colored dashed lines. Red, H-bond; orange, polar interactions; yellow, ionic interactions; cyan, van der Waals; light green, aromatic interactions; dark green, hydrophobic interactions.

During the oncological evaluations of a Ukranian 65-year-old woman affected by TNBC, we identified the *BRCA1* c.5057A>C (p.His1686Pro) VUS. The missense alteration here reported is located in the exon 16 of the *BRCA1* gene and leads to a histidine-to-proline substitution at amino acid position p.1686 with a variant allele frequency of 46%. The same molecular alteration was detected in the daughter affected by adenocarcinoma of the uterine cervix (35 years old) and in a niece affected by BC (41 years old). We were not able to perform *BRCA* evaluation in the other alive BC affected nieces that declined to be part of the study. Moreover, the deceased brother affected by pancreatic cancer and niece affected by BC did not perform *BRCA* genetic test. Blood samples from other family members were not available for the analysis (**Figure 1**). The *BRCA1* c.5057A>C (p.His1686Pro) variant is annotated in the main public database as a rare VUS (rs730882166). In particular, it is classified as VUS in ClinVar repository (https://www.ncbi.nlm.nih.gov/clinvar/variation/584509/?new_evidence=true, accessed 24 September 2022) with a single submitter (August 2018) and as unclassified variant in LOVD database (https://databases.lovd.nl/shared/variants/0000627446#00003478, accessed 24 September 2022). It does not have a gnomAD genomes entry (https://www.gnomad.broadinstitute.org/, accessed 24 September 2022) or annotation in BIC and *BRCA* Exchange databases, confirming the rarity of the alteration. This alteration was never found among all the large cohort of subject (about 1,000/years) underwent *BRCA* testing in our Institution since 2014. Given the unknown significance of the *BRCA1* c.5057A>C (p.His1686Pro) variant and to exclude the presence of others genetic drivers of disease, we performed an extended multi-gene analysis evaluation on both the proband and her daughter, excluding the co-occurrence of other high-risk mutations in cancer-related genes. To investigate about the effect of the variant, we set out an alignment of multiple BRCA1 protein sequences showing that the His1686 residue is extremely conserved among species and lies in a key protein domain, the THV motif of the BRCT repeat (**Figure 2**). Moreover, the bioinformatic evaluation of the *BRCA1* variant performed using VARSOME *in silico* tool revealed a disease causing effect of the nucleotide substitution A>C, with a final verdict of pathogenicity: 17 of the 18 individual prediction with scores of damaging impact on protein function or structure. In addition, a high probability of pathogenicity resulted from PRIOR prediction. InterVar clinical interpretation classified the variant as likely pathogenic according to the ACMG/AMP guideline. Evaluation of the protein stability as in the DynaMut prediction underlines that substitution of the His1686 residue with Pro1686 has a destabilizing effect on the 3D structure of the protein as it is witnessed by the computed ΔΔGStability of −1.38 Kcal/mol. Tridimensional structures of the wild-type (A) and mutant (B) protein as in **Figure 3** depict the mutant protein as being concerned in a richer array of interactions with the neighbor amino acid residues as compared with the wild-type counterpart. In turn, this indicates a higher flexibility of the wild-type protein and, thus, a better propensity to PPI when compared to the single substitute version of BRCA. Nevertheless, the prediction of the PPI as in the two independent algorithms targeting the protein sequences (i.e., PSOPIA and Tri-Tool) disagrees with observations recorded in the above featuring of the whole-protein structure.

Prediction of the PPI for the wild-type and mutant protein against a putative list of interacting proteins is summarized in **Table 1**. Both mutant and wild-type proteins are predicted to have the same propensity of interaction with the list of selected target proteins. Predicted PPI scores are indeed the same in all prediction criteria when comparing BRCA1 wild type and BRCA1 p.(His1686Pro), suggesting that a single-point mutation in these protein sequences is not sufficient to fairly underline its phenotypic consequences. Prediction of PPI as in the Tri-Tool algorithm is summarized in **Table 2**. Interestingly, observed PPI predictions are in line with the above data from PSOPIA prediction. Here, very minor differences are observed while comparing PPI predictions of pairs with the BRCA1 against the predictions calculated for the pairs with BRCA1 p.(His1686Pro). Considering these observations, we concluded that the effect of a single-mutation imprints a structural change at the whole-protein structure level that, in turn, is suggestive of a diverse interaction propensity of the mutant and wild-type proteins. Although, the prediction of the PPI as in the PSOPIA and Tri-Tool portraits a similar interaction propensity for both proteins. Here, both PPI algorithms work independently with different approaches, as reported in above; however, it must be bear in mind that both algorithms focus on the sole protein sequences and analyze the single–amino acid composition of the two proteins, missing to consider the whole-protein structure that is most likely the feature responsible for the diverse array of interaction that the two proteins are capable of.

**Table 1:** Protein–protein interaction (PPI) prediction by PSOPIA.

	Gene name	UniProt ID	Sequence score	Domain score	Network score	Overall score
**BRCA1_ *Wild Type***	***BRCA2***	P51587	0.3537	0.5146	0.4292	0.3497
	***UIMC1***	Q96RL1	0.3537	0	0	0.3537
	***ABRAXAS1***	Q6UWZ7	0.3537	0	0	0.3537
	***ACACA***	Q13085	0.3537	0.5146	0.1324	0.1015
	***BRIP1***	Q9BX63	0.3537	0.5146	0.4292	0.3497
	***CCAR2***	Q8N163	0.3537	0	0	0.3537
	***LMO4***	P61968	0.5333	0.3897	0.8351	0.2924
	***MSH2***	P43246	0.3613	0.5146	0.4292	0.3525
	***RBBP8***	Q99708	0.813	0.5146	0.8351	0.9011
	***BACH1***	O14867	0.3722	0.3897	0.6359	0.2721
	***MAP3K3***	Q99759	0.5333	0.3897	0.8351	0.2924
	***AURKA***	O14965	0.5333	0.3897	0.8351	0.2924
**BRCA1_ p.His1686Pro**	***BRCA2***	P51587	0.3537	0.5146	0.4292	0.3497
	***UIMC1***	Q96RL1	0.3537	0	0	0.3537
	***ABRAXAS1***	Q6UWZ7	0.3537	0	0	0.3537
	***ACACA***	Q13085	0.3537	0.5146	0.1324	0.1015
	***BRIP1***	Q9BX63	0.3537	0.5146	0.4292	0.3497
	***CCAR2***	Q8N163	0.3537	0	0	0.3537
	***LMO4***	P61968	0.5333	0.3897	0.8351	0.2924
	***MSH2***	P43246	0.3613	0.5146	0.4292	0.3525
	***RBBP8***	Q99708	0.813	0.5146	0.8351	0.9011
	***BACH1***	O14867	0.3722	0.3897	0.6359	0.2721
	***MAP3K3***	Q99759	0.5333	0.3897	0.8351	0.2924
	***AURKA***	O14965	0.5333	0.3897	0.8351	0.2924

The table summarizes the PPI predictions performed by PSOPIA bioinformatic tool. For each pair of protein, the score of PPI is computed on the basis of the sole protein sequence (sequence score column), domain of interactions (domain score column), and network of interactions (network score column), and the averaged PPI prediction is computed on the basis of all the previous parameters (overall score column). PPI score is a value ranging from 0 and 1, where 1 represent the maximum interaction propensity of a protein pair.

**Table 2:** Protein–protein interaction (PPI) prediction by Tri-Tool.

	Target protein	Probability
**BRCA1_*Wild Type***	Q99759	0.65
	Q96RL1	0.81
	Q6UWZ7	0.68
	Q13085	0.74
	Q9BX63	0.73
	Q8N163	0.57
	P61968	0.78
	P43246	0.73
	Q99708	0.79
	O14867	0.76
	O14965	0.51
**BRCA1_p.His1686Pro**	Q99759	0.65
	Q96RL1	0.80
	Q6UWZ7	0.67
	Q13085	0.73
	Q9BX63	0.72
	Q8N163	0.57
	P61968	0.78
	P43246	0.72
	Q99708	0.78
	O14867	0.76
	O14965	0.51

The table lists the PPI predictions calculated for each of the pair BRCA1-target proteins and BRCA1 p.His1686Pro-target proteins. PPI prediction is expressed as the probability (P) of a given pair to form an interaction. P > 0.5 is assumed to be a valid interaction.

## 4 Discussion

In the present paper, we discussed the molecular strategy characterization adopted for the evaluation of a *BRCA1* VUS identified in a patient affected by TNBC. We aim to underline the relevance of the adoption of tailored VUS analysis, integrated in the routine practice, for the proper management of patients. Overall, the in-depth assessment of the *BRCA1* p.(His1686Pro) VUS posed several pieces of evidence for its pathogenicity significance. Moreover, it reflected how important is improving the collection of clinical information, family history, sequencing, and molecular data in the real-life clinical setting. In this way, we were able to provide to the patient and relatives an enhanced counseling with a quick monitoring asset, increasing awareness around the test.

Germline *BRCA* mutations confer a well-established increased risk for the development of all BC subtypes, with TNBC mainly associated to *BRCA1* defects (8). Germline *BRCA* testing in BC-affected patients was previously used mainly to guide surgical approaches. To date, the clinical relevance of *BRCA* testing in such patients lies in the possibility to identify BC cases who might benefit from additional or alternative treatment options, as well as surveillance strategies (21–24). Patients with advanced or metastatic germline *BRCA*-mutated BC show high response rate to platinum salts (i.e., cisplatin and carboplatin). Moreover, poly (ADP-ribose) polymerase inhibitors (PARPi) have received Food and Drug Administration (FDA) approval for the treatment of germline *BRCA*-mutated HER2-negative metastatic BC previously treated with chemotherapy in neoadjuvant, adjuvant, or metastatic settings. In this scenario, most of the pre-clinical and clinical data derived from *BRCA*-mutated carriers with TNBC (8, 21, 22). The observation that TNBC are frequently *BRCA1*-mutated, with a gene expression profiles similar to others *BRCA1*-deficient tumors (22), represented one of the first molecular insights for this type of cancer. Overall, LoF mutations in *BRCA* genes are present in up to 20% of patients with TNBC.

In this context, a consistent percentage of BC families that underwent *BRCA* gene testing experienced uninformative results due to the identification of gene variants with unknown biological and clinical significance, i.e., VUS (25). While an overall VUS rate of 7%–15% in women who have received *BRCA* testing has been reported, the frequency of VUS varies worldwide depending on the testing prevalence and population ancestry. Researchers reported a frequency of VUS of 21% in African–Americans, 5%–6% in people of European ancestry in the United States, and 15% in European laboratories (26). In these cases, the clinical management concerning prophylactic surgery and therapeutic strategies depends entirely on the family cancer history and on other risk factors evaluation, pending a reclassification of the detected variant. Incorrect classification of a variant as likely pathogenic can lead to a patient’s “over-management”. Conversely, significant consequences of variants under classification can occur with interventions and therapies administration not appropriate to the true level of risk (27). Limited studies investigating the impact of *BRCA* VUS on clinical decision-making are available in the literature. Some of these reported comparable mastectomy rates between patients with *BRCA* wild type and *BRCA* VUS (28). Others authors highlighted intermediated rates, higher than *BRCA* wild-type and lower than *BRCA*-mutated patients (29). However, it is challenging to understand whether the prophylactic surgery was adopted according to a combination of VUS identification with other factors, e.g., family history or patient’s distress.

To date, the widespread use of NGS high-throughput technologies allows the screening of thousands of affected individuals, leading also to the identification of an increasing number of VUS. Currently, ClinVar public database has registered about 5,200 *BRCA1* germline variants. Approximately 80% of pathogenic or likely pathogenic variants leads to immature stop codons and encoded protein truncation, reducing their expression *via* nonsense-mediated mRNA decay (NMD). *BRCA1* missense mutations mainly involved the RING and C-terminal tandem BRCT domains. Among the other annotated missense variants, there are approximately 1,300 VUSs (30). In the era of personalized medicine, the identification of a VUS prompts clinicians and geneticists to argue about the best strategy for guarantee and maximize standards of care. In our Institution, the establishment of Tumour Molecular Board is aimed to an in-depth evaluation and clarification of patients’ molecular result in view of clinical setting and increases the consciousness about VUS management among geneticists and non-geneticist clinicians.

Here, we described our strategy adopted in a typical case of BC-affected woman carriers of a *BRCA* VUS. In our clinical context, this approach is adopted in all relevant cases with a strong family history of cancer related to *BRCA* genes, and it is of great support for several reasons: 1) it improves patient’s clinical management, even if not directly impact on therapeutic path but through a stronger monitoring of patient and relatives; 2) it adds valuable information about VUS in clinical setting, making possible future targeted strategies; 3) it favors the multidisciplinary management of patients, involving laboratory experts in multiple fields, oncologists, and geneticists; 4) it allows in the long-term to boost awareness of VUS role and appropriate understanding of their relevance.

Our argumentation underlines that the efficient integration of clinical data with updated molecular databases and predictive algorithms is crucial for making VUS eligible for clinical use.

As here reported, the *BRCA* testing revealed the presence of the unclassified *BRCA1 c.5057A>C* (p.His1686Pro) variant in the BC affected proband, in her daughter affected by an adenocarcinoma of the uterine cervix, and in a BC affected niece. According to the described strategy, several lines of evidence supported the likely pathogenic role of the *BRCA1* p.(His1686Pro) variant. With regard to this, the Working Group on Unclassified Sequence Variants of the International Agency for Research on Cancer (IARC) defined the types of evidence that measure a more “direct” association of the variant with the disease and other “indirect” evidence linked to the observed or predicted effect of the variant on gene structure and function (31). Traditionally, VUSs in genes conferring Mendelian risk of disease have been classified as disease-associated using co-segregation analyses in multiple affected families. However, it is rarely possible to classify a VUS based on this direct genetic evidence alone, due to the low number of accessible cases in small pedigrees and the unavailability of genetic materials belonging from different affected subjects (12). Moreover, considering that the cancer risk associated with VUS is unknown, co-segregation analysis is rarely performed in these families (32). With regard to this, the *BRCA1* p.(His1686Pro) alteration was recently reported in only another family with BC (ClinVar accession number VCV000584509.3, August 2018).

In addition, high allele frequency in healthy individuals is considered a strong indicator for a benign interpretation (33). Regarding case-control association, the *BRCA1* p.(His1686Pro) variant does not have a gnomAD or 1000 Genomes entry, suggesting that this is a very rare *BRCA1* alteration. In addition, we can define the *BRCA1* p.(His1686Pro) variant as rare variants also on the basis of our large cohort of subjects that we screened so far in our institution.

The most common and direct approach to speculate about the pathogenicity of a variant is the personal and family history analysis. In the case of *BRCA1* c.5057A>C (p.His1686Pro), we reported a strong association to cancer-affected members of the family as reported above. Unfortunately, despite the cooperation of the family members, it was not possible to perform the molecular analysis in all the proband’s alive relatives due to the unavailability of the blood samples or their refusal to participate in the study. We underlined that it was challenge for us to collect complete molecular and clinical data of the family members who live in Ukraine that also experienced limited access to the molecular analysis.

In addition, to exclude the presence of another high-risk mutation in the family, we extended the genetic evaluation to 26 hereditary cancer-associated genes. In fact, the co-occurrence of a PV in the affected patients could be clearly related to the disease occurrence, and it could be the main cause of disease aggregation. Both the affected proband and her daughter resulted wild type for other PVs or VUS in the investigating genes.

Indirect evidence of pathogenicity may be derived from specific features of the variant including structural information about the gene and the protein, functional ability of the mutated protein, and the *in silico* analysis of the mutated sequence. The His1686 residue showed a high interspecies conservation of the amino acid at the substitution site, suggesting that this amino acid might be relevant for BRCA1 structural/functional correlation. In particular, the His1686 residue belongs to a THV motif involved in the formation of a groove on the N-BRCT surface opposite to the cleft involved in phosphoepitope binding (34). For *BRCA1*, most clinically relevant mutations reside in the well-characterized N-terminal RING finger domain and C-terminal tandem BRCT repeats (35–37). The BRCT domain is a phosphoprotein-binding module that mediates the interaction with several BRCA1 functional partners, such as BRIP1/FANCJ, CtIP, and Abraxas, and it plays a critical role in DNA damage response and repair processes (38). Moreover, BRCT domains contain several BC-prone regions associated with a relative excess of breast vs. ovarian cancers (Ratio of breast vs ovarian cancer Hazard Ratio (RHR) = 1.38; 95% CI, 1.22–1.55; P = 6 × 10−9) (39). Given these crucial aspects related to the BRCT domain, it can be assumed that alterations in amino acid sequence lead to relevant consequences in protein structure and/or function. Overall, from our *in silico* analysis emerged as the *BRCA1* p.(His1686Pro) variants is predictive to have a damaging impact of protein function.

In addition, we evaluated the effect of the missense variant on BRCA1 protein stability and dynamics using a web-based approach. *In silico* evaluation of the protein stability in its wild-type and mutant version, based on the available three-dimensional structure, portraits a higher stability of the *BRCA1* when compared to its mutated counterpart. Moreover, the higher number of intra-molecular interactions predicted for the wild-type protein suggests *BRCA1* wild-type as a much more flexible molecule than *BRCA1* p.(His1686Pro), thus capable of interaction with a wider plethora of molecules. In this scenario, a computer-aided prediction of the PPIs against a list of putatively interacting proteins selected from literature data (17, 18) disregarded our thoughts, underlining highly similar PPI trends for the wild-type and point mutant proteins. Acknowledging the diverging results from our initial expectations, we might argue that PPIs have been computed on the whole-protein sequences and that it is likely that the effect of a point mutation over a long protein sequence (of 1,863 amino acid residues) might not be sufficient to score different PPI trends for the BRCA1 and BRCA1 p.(His1686Pro). The reasons for the above might be technical and/or biological; in the first instance, we assume that this point mutation is effectively driving a different PPI trend among the two protein versions, but its effect is masked by the remaining amino acidic sequence that is identical in both proteins, preventing the algorithm from discriminating the different PPI trends. On the other hand, it is also plausible that changes in the twist forces imputed to the point mutation is not sufficient to transmit such alteration to the whole molecule; thus, it is not altered the interaction propensity of the molecules, although the phenotypic effect is preserved (40). Finally, the list of putatively interacting proteins represents a limited subset of molecule selected among hundreds to be part of *BRCA* pathway.

Furthermore, supporting evidence emerged from literature data regarding the protein functionality and the annotation of variants accounted in the same nucleotide/codon position or in the surrounding gene region. Findlay et al. performed a systematic classification of the *BRCA1* missense variants applying saturation genome editing to measure the functional consequences with more than 95% accuracy (41). The authors defined for the *BRCA1* p.(His1686Pro) a function score of −1.97, corresponding to a functional classification of the variant as LoF. Moreover, a large study aimed to the integration and the harmonization of available functional data provided evidence in favor of pathogenicity for the *BRCA1* p.(His1686Arg) variant, with a high level of strength (42). In addition, the *BRCA1* p.His1686 amino acid position is involved in several residue changes previously associated to BC and *BRCA*-related cancers. The *BRCA1* p.(His1686Arg) variant was annotated as pathogenic in LOVD and ClinVar repository (VCV000584509.3) in association with several patients with BC and ovarian cancer. These classifications resulted from clinical and functional studies that proved its damaging effect (41, 43, 44). The same codon was involved in the *BRCA1* p.(His1686Gln) missense variant reported as pathogenic in association with BC development (9). Additional observations belonged from the pathogenic role of the *BRCA1* missense variants p.(Thr1685Ile) (ClinVar, accession number VCV000055365.6, last accessed 24 September 2022), p.(Thr1685Ala) (ClinVar, accession number VCV000055364.13, last accessed 24 September 2022), and p.(Val1687Gly) (ClinVar, accession number VCV000433721.5, last accessed 24 September 2022) that prove the structural relevance of this motif within the BRCA1 BRCT domain.

Finally, the identification of *BRCA* VUS still represents a rate-limiting step of the gene testing. As case in point, we provided multiple findings that could support the interpretation of the p.(His1686Pro) missense mutation as *BRCA1* pathogenic variant in appropriate clinical contexts. From our point of view, the study of the pathogenic role of uncertain *BRCA* variants represents a priority for the management and care of the affected as well as high-risk subject who receive uninformative genetic result.

A limitation of the present study is the unavailability of *in vitro* functional assays investigating the effect of the p.(His1686Pro) variant, although several functional data emerged from literature. Variants functional annotations are overall a prerequisite for gene-disease relationship analysis and play an indispensable role in final classification. We want to underline that BRCA1 is a large and complex protein with multiple functions in several processes (30, 44, 45). Loss of a specific activity due to the occurrence of a single–amino acid substitution is not easily demonstrable, and it is also difficult to clinically interpret. Consequently, several functional assays should be integrated and interpreted in light of clinical and familial data. This approach is generally not in compliant with clinical purposes, against the even-increasing number of VUS identified in the era of NGS and tumor molecular profiling. The precise functional annotation of VUS derives from labor-, time-, and resource-consuming biochemical assays while, at the same time, the discovery of VUS is in faster speed than the carryout of biochemical experiments. Consequently, the evaluation of VUS clinical significance should therefore preferably rely mainly on clinical data, supported by molecular evidence, which is directly related to disease risk (32).

In conclusion, the accurate assessment of VUS relevance in the specific clinical context is critical, and ongoing efforts are needed to collect and share the widest number of supporting evidence aimed to solve the molecular and clinical gap involving uncertain variants. We support the active involvement of expert’s panels of clinicians, molecular biologists, pathologists, geneticists, and bioinformatics to optimize data integration about “orphan” *BRCA* variants and to share the relevant evidence.

## Data availability statement

The original contributions presented in the study are included in the article/supplementary material. Further inquiries can be directed to the corresponding author.

## Ethics statement

The studies involving human participants were reviewed and approved by Ethics Committee of Fondazione Policlinico Universitario “A. Gemelli” in Rome. The patients/participants provided their written informed consent to participate in this study.

## Author contributions

EP, IP, BT, AM, and CS conceived of the presented paper and wrote the first draft of the manuscript. EP, LF, and BT supported the experimental steps. IP, PR, DG, and AU supervised the project and revised the final manuscript. All authors discussed, edited, and contributed to the final manuscript, approving the submitted version.
